# Multiple Listeria Abscesses in an Immunocompetent Patient

**DOI:** 10.7759/cureus.6642

**Published:** 2020-01-13

**Authors:** Heitor C Frade, Chandra Pingili, Premkumar Nattanamai

**Affiliations:** 1 Neurology, University of Missouri, Columbia, USA

**Keywords:** listeria monocytogenes, brain abscess, central nervous system infections

## Abstract

Listeria monocytogenes is a ubiquitous organism that can potentially cause gastroenteritis and, less commonly, central nervous system infections. Brain abscess is rare and often associated with immunocompromised status. We report a case of multiple abscesses caused by Listeria in a previously immunocompetent elderly patient who developed a headache and left-sided hemiparesis over the course of days. Neuroimaging studies revealed multiple ring-enhancing lesions in the brain and midbrain territories. Blood culture, brain tissue aspirate, and cerebrospinal fluid nucleic acid amplification test were positive for Listeria. Extensive immunologic workup revealed no primary or secondary immunodeficiency disorders. After the initiation of antibiotics, the patient showed gradual clinical improvement and went to a skilled nursing facility after two weeks.

## Introduction

Listeria monocytogenes is a foodborne pathogen that most commonly causes gastroenteritis but may also affect the central nervous system (CNS), particularly in newborns, pregnant women, elderly and immunosuppressed patients. It is estimated that 1,600 new cases of listeriosis occur every year in the United States [1]. Listeria reach the CNS via hematogenous spread by crossing blood-brain and blood-choroid barriers. Although the most common CNS manifestation is meningitis, which accounts for 20% of bacterial meningeal infections in both neonates and elderly patients, it may also present as encephalitis, rhombencephalitis, and brain abscess [2,3].

Brain abscesses account for less than 10% of CNS listerial infections. They are mostly singular lesions, located on cortical or subcortical regions. When symptomatic, common findings include fever, mental status changes and focal neurological deficits. Bacteremia is often present, in comparison to other bacterial brain abscesses, while cerebrospinal fluid (CSF) cultures were positive in less than half of cases [4,5]. Neuroimaging (CT or MRI) may show focal lesions and/or hydrocephalus.

Although the management of most abscesses requires surgical drainage, antibiotic therapy alone may be successful in treating listerial abscess. The preferred antilisterial agent is ampicillin, sometimes combined with aminoglycosides. If the patient has penicillin hypersensitivity, trimethoprim/sulfamethoxazole is a reasonable alternative [3]. The condition may recur after successful treatment, with or without surgical intervention.

Prognosis may vary according to the presence of risk factors, particularly immunosuppression. Neurological sequelae are common, which depends on the severity and location of the lesion. Mortality is higher than in patients with nonlisterial abscess, but there seems to be no significant difference in mortality between patients with single or multiple listerial abscesses, neither between patients treated with monotherapy or combination therapy [6,7].

## Case presentation

A 74-year-old female presented to an outside hospital (OH) after a four-day history of headache and a recent fall. Her past medical history was significant for migraines, post-traumatic seizure disorder treated with levetiracetam, coronary artery disease, and supraventricular tachycardia managed with amiodarone and diltiazem. There was no history of alcohol or recreational drug use, but the patient was a chronic smoker. The patient had no focal neurological deficits on presentation to the OH, consistent with a CT head (CTH). Although the patient showed no abnormalities on admission to OH, she developed left-sided hemiparesis over the course of that day. A second CTH performed on the afternoon of the admission was consistent with right basal ganglia hypodensity, so the patient was started on single antiplatelet therapy. Further imaging assessment with MRI showed increased T2 and fluid -attenuated inversion recovery (FLAIR) signals involving a subcortical right hemisphere lesion suggestive of glioma, which motivated a transfer to our hospital.

**Figure 1:**
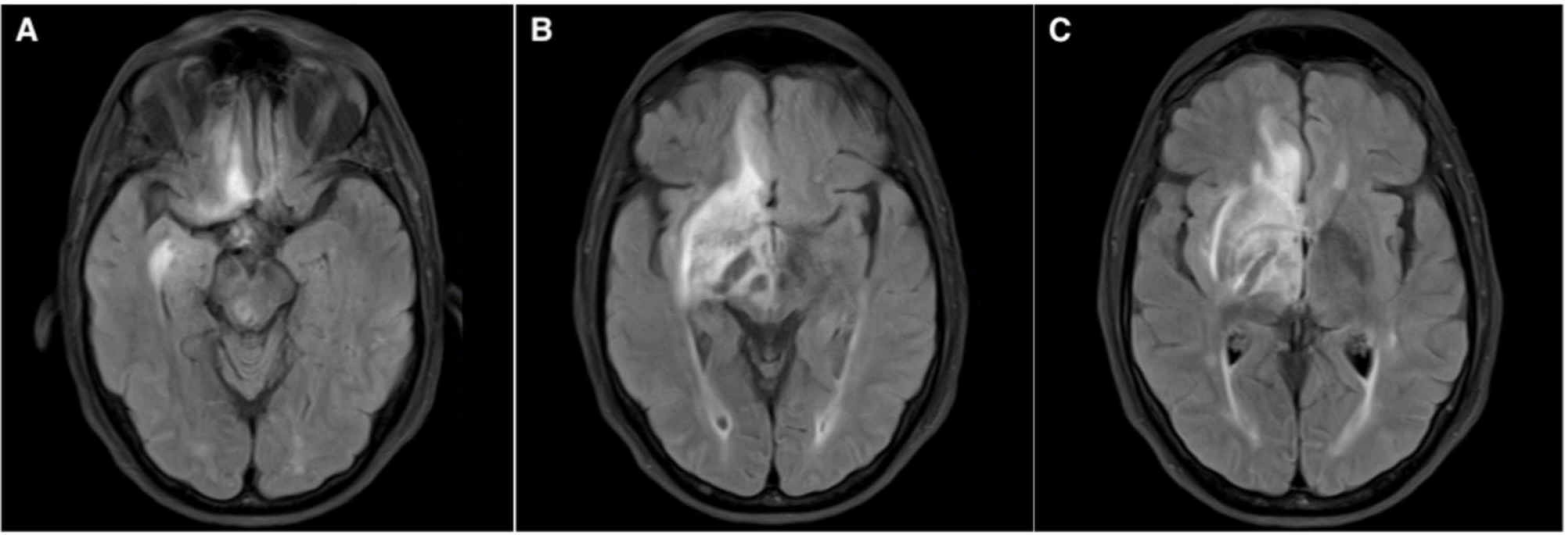
Brain MRI on admission FLAIR MRI sequence on axial plane showing focal hyperintensities in the midbrain (A), basal frontal and temporal lobes (A, B), thalamus and basal ganglia (C) FLAIR, fluid attenuated inversion recovery

On admission to our hospital, patient was afebrile, eupneic, alert and oriented, with neurological examination revealing paresthesia and mild weakness (4/5) on left upper and lower extremities. Cranial nerve examination and plantar response testing showed no abnormalities. Laboratory examinations initially showed iron-deficiency anemia and mild hyponatremia (Table 1). A repeat brain MRI without contrast showed similar findings to previous imaging, i.e. T2/FLAIR hyperintense lesions in the right basal frontal and temporal lobes, thalamus, and brainstem (Figure 1).

**Table 1:** Laboratory findings on admission WBC, white blood cell; TIBC, total iron-binding capacity; BUN, blood urea nitrogen; ALP, alkaline phosphatase; AST, aspartate aminotransferase; ALT, alanine aminotransferase

Hematologic profile	Value	Metabolic panel	Value
Hemoglobin (g/dL)	9.8	Sodium (mmol/L)	131
Hematocrit (%)	31.2	Potassium (mmol/L)	4.1
Platelets (1,000/µL)	347	Chloride (mmol/L)	96
WBCs (1,000/µL)	5.38	Bicarbonate (mmol/L)	22
% Neutrophils	80.9%	BUN (mg/dL)	16
% Immature granulocytes	0.7%	Creatinine (mg/dL)	0.4
% Lymphocytes	14.5%	Glucose (mg/dL)	156
% Monocytes	3.7%	Calcium (mg/dL)	8.3
% Eosinophils	0.0%	Magnesium (mg/dL)	2.1
Serum iron (mcg/dL)	17	ALP (u/L)	65
TIBC (mcg/dL)	365	AST (u/L)	18
Ferritin (ng/mL)	23.9	ALT (u/L)	18

**Figure 2:**
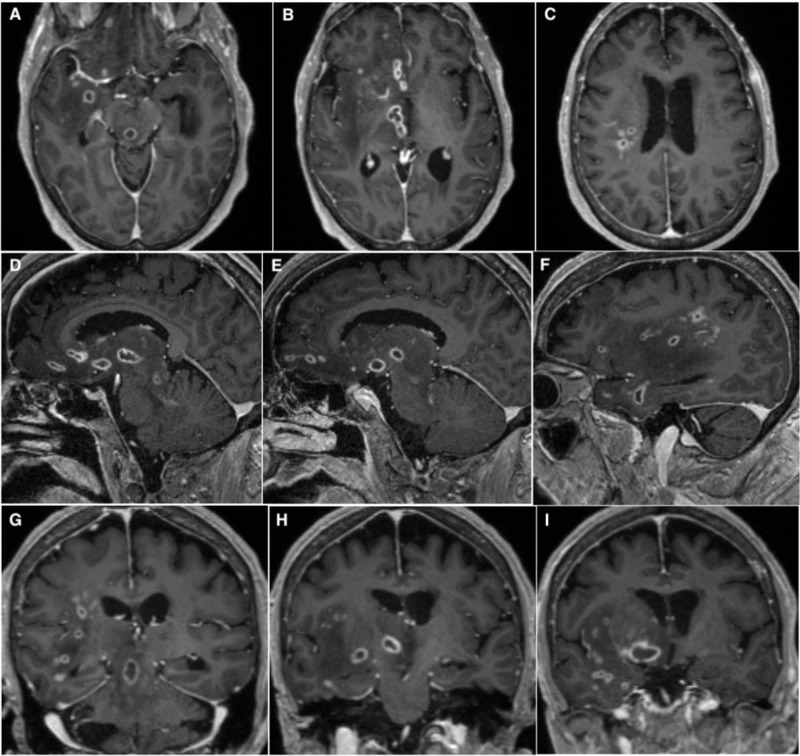
Contrast-enhanced MRI Contrast-enhanced MRI T1 sequence on axial (A-C), sagittal (D-F), and coronal (G-I) planes showing unilateral ring-enhancing lesions in the midbrain, thalamus, and frontotemporal subcortical regions.

**Figure 3:**
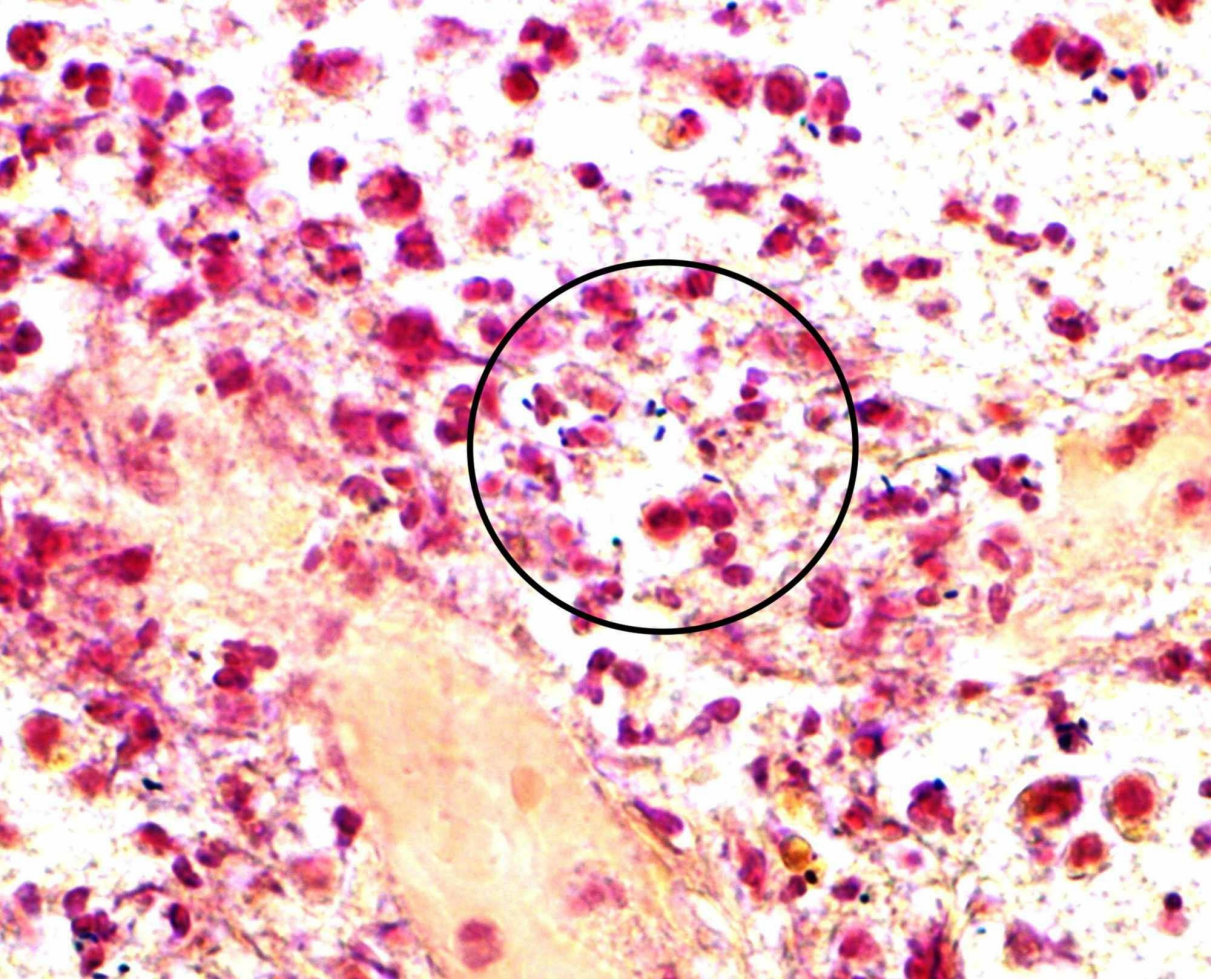
Gram stain of brain biopsy specimen Brain biopsy sample with X60 magnification showing gram-positive rods

On hospital day 5, the patient underwent stereotactic biopsy of a lesion located in the right frontal lobe, followed by high-volume lumbar puncture. After the procedure, severe hemiparesis (1/5) and a first episode of fever (38.4°C) were noted, and the following labs on hospital day 6 revealed leukocytosis (12,580/µL) with neutrophilic predominance (84%). CSF analysis revealed elevated protein and lactate, as well as mild lymphocytic pleocytosis (Table 2), while biopsy revealed signs of acute inflammation and abscess. Follow-up MRI with contrast revealed multiple ring enhancing lesions in right basal frontal and temporal lobes, thalamus, and midbrain (Figure 2). Under the suspicion of septic metastases, empiric treatment was initiated with vancomycin, cefepime, and dexamethasone. After a CSF BioFire® meningitis/encephalitis (ME) panel (BioFire Diagnostics, a bioMérieux Company, Salt Lake City, UT) returned positive for Listeria, antibiotics were switched to ampicillin 12 g/day and metronidazole 1.5 g/day. Although the CSF culture was negative, a blood culture was positive for Listeria sensitive to ampicillin and sulfamethoxazole-trimethoprim (Table 3), so metronidazole was discontinued. Seven days after biopsy, culture returned positive for gram-positive organisms consistent with Listeria (Figure 3).

**Table 2:** Cerebrospinal fluid analysis HD, hospital day

	HD 5	HD 8	HD 13
Leukocytes (/µL)	41	435	159
Neutrophils (%)	-	22	3
Lymphocytes (%)	100	78	97
Protein (mg/dL)	65	202	82
Glucose (mg/dL)	51	83	84
Lactate (mg/dL)		66.6	

**Table 3:** Cultures and BioFire® meningitis/encephalitis (ME) panel HD, hospital day; CSF, cerebrospinal fluid

	HD 5	HD 6	HD 7	HD 8	HD 13
Blood culture	+	-	-		
CSF culture	-			-	-
CSF BioFire® ME panel	+				
Brain tissue culture	Gram + cocci				

On hospital day 12, the patient developed a lumbar rash consistent with zoster, but skin swab test later revealed herpes simplex virus 2 (HSV-2). A repeat CSF analysis on hospital day 13, eight days after antibiotics were started, was negative for Listeria but positive for HSV-2, consistent with the concurrent skin biopsy findings, so acyclovir was added to the regimen. Due to the concern for immunodeficiency, the patient was tested for HIV, human T-lymphotropic virus, syphilis, viral and autoimmune hepatitis, hypogammaglobulinemia, and hypocomplementemia, but all the investigations came out to be negative. Flow cytometry revealed a CD4 count of 253 cells/µl. CT and MRI abdomen done for hidden neoplasms revealed hepatic cysts, and serum biomarker screening showed elevated serum carcinoembryonic antigen and cancer antigen 125.

At the time of discharge, on hospital day 22, patient was conscious and oriented, with neurological examination remarkable only for severe left-sided weakness (1/5) attributed to initial progression of the infection and surgical manipulation. She was transferred to a skilled nursing facility with instructions to complete antibiotic regimen for a total of six weeks, and with scheduled follow-up visits for further assessment of her hepatic condition.

## Discussion

Listeria is a facultative intracellular bacterium mostly associated with foodborne illness, but it also can cause CNS infection, neurolisteriosis, particularly in the setting of impaired cell-mediated immunity. Neurolisteriosis commonly affects the meninges, but it may also affect brain parenchyma. Subcortical areas, thalamus, pons, and medulla are usual targets [8]. In this patient, there were multiple ring-enhancing lesions suggestive of abscesses in right frontotemporal subcortical areas, thalamus, and midbrain. More than one foci have been described in only 25% of patients with listerial abscesses [6].

This patient had a gradual course of worsening headache and focal findings, the left-sided weakness, and paresthesia, all of which are congruent to a CNS abscess rather than meningitis. There were no fever, nausea, vomiting, or diarrhea reported before or after admission to our hospital. Blood tests revealed mild leukocytosis, which was aligned with the previous reports [9,10]. CSF analysis showed elevated levels of lactate, which has been reported as a useful diagnostic marker to differentiate between viral CNS infections and Listeria or other bacterial infections [11].

In microbiology studies, we found positive blood culture associated with negative CSF culture, consistent with the previous findings [7]. Our CSF was further analyzed with a nucleic acid amplification test (NAAT), a BioFire® ME panel, which was positive for Listeria DNA, and tailored our empiric antibiotic regimen to cover Listeria. This suggests an important role for NAAT, as a faster and considerably sensitive alternative for ruling in Listeria before culture results are available, potentially improving morbidity and mortality.

Abscess is particularly rare among neurolisteriosis presentations and is often related to immunosuppression. In a review of 39 cases of Listeria abscesses, 85% of patients were immunocompromised by HIV infection, lymphoma, leukemia, or conditions requiring chronic immunosuppressive therapy [4]. Our patient initially presented with no evidence of immunosuppressive treatment or condition, but workup revealed hepatic lesions and elevated tumor markers concerning for malignancy. These findings could represent a malignancy contributing in some degree to immune suppression, although there was no confirmatory evidence by the time the patient was discharged. Also, during her inpatient course, she developed skin lesions secondary to HSV-2 reactivation. Even though herpesvirus reactivations suggest immune suppression, they may also be related to normal aging [12].

Our patient showed clinical improvement after empiric and sensitivity-guided antibiotic regimens for Listeria, but still retained left-sided weakness by the time of discharge, likely secondary to both abscess burden and brain surgery. Timely initiation of empiric antibiotics has been associated with better clinical outcome [13]. For better diagnostic accuracy and antibiotic coverage, an empiric regimen should be started shortly after the aspiration of one abscess has been performed, and should be able to cover staphylococci, streptococci, strict anaerobes, and Enterobacteriaceae [14].

Multiple abscesses by Listeria are a rare presentation of neurolisteriosis and have been mostly reported in immunosuppressed adult or elderly patients [6]. Although there was no confirmatory evidence of immune suppression in this patient by the time she was discharged, there were scheduled follow-up appointments for further assessment of the recently found hepatic cystic lesions.

## Conclusions

We reported an atypical presentation of CNS listerial infection in a previously immunocompetent patient. Despite initially non-focal symptoms, further testing revealed multiple listerial abscesses. After initiation of antibiotics, this patient had a good response and a favorable short-term outcome. Although rare in immunocompetent patients, it is important to consider Listeria in the differential diagnosis of single or multiple brain abscesses.
